# Beyond vegetables: effects of indoor LED light on specialized metabolite biosynthesis in medicinal and aromatic plants, edible flowers, and microgreens

**DOI:** 10.1002/jsfa.11513

**Published:** 2021-09-15

**Authors:** Elisa Appolloni, Giuseppina Pennisi, Ilaria Zauli, Laura Carotti, Ivan Paucek, Stefania Quaini, Francesco Orsini, Giorgio Gianquinto

**Affiliations:** ^1^ DISTAL – Department of Agricultural and Food Sciences Alma Mater Studiorum University of Bologna Bologna Italy; ^2^ FEEM—Foundation Eni Enrico Mattei Milan Italy

**Keywords:** horticultural light emitting diodes, indoor farming, vertical farms, secondary metabolites, nutraceuticals, antioxidant

## Abstract

Specialized metabolites from plants are important for human health due to their antioxidant properties. Light is one of the main factors modulating the biosynthesis of specialized metabolites, determining the cascade response activated by photoreceptors and the consequent modulation of expressed genes and biosynthetic pathways. Recent developments in light emitting diode (LED) technology have enabled improvements in artificial light applications for horticulture. In particular, the possibility to select specific spectral light compositions, intensities and photoperiods has been associated with altered metabolite content in a variety of crops. This review aims to analyze the effects of indoor LED lighting recipes and management on the specialized metabolite content in different groups of crop plants (namely medicinal and aromatic plants, microgreens and edible flowers), focusing on the literature from the last 5 years. The literature collection produced a total of 40 papers, which were analyzed according to the effects of artificial LED lighting on the content of anthocyanins, carotenoids, phenols, tocopherols, glycosides, and terpenes, and ranked on a scale of 1 to 3. Most studies applied a combination of red and blue light (22%) or monochromatic blue (23%), with a 16 h day^−1^ photoperiod (78%) and an intensity greater than 200 μmol m^−2^ s^−1^ (77%). These treatment features were often the most efficient in enhancing specialized metabolite content, although large variations in performance were observed, according to the species considered and the compound analyzed. The review aims to provide valuable indications for the definition of the most promising spectral components toward the achievement of nutrient‐rich indoor‐grown products. © 2021 The Authors. *Journal of The Science of Food and Agriculture* published by John Wiley & Sons Ltd on behalf of Society of Chemical Industry.

## INTRODUCTION

Unlike primary metabolites involved in the basic metabolic functions and development of all plants, secondary or specialized metabolites are not essential for plant life, and their distribution and biological effects can vary between species depending on the specific roles that they play.^1^ Nevertheless, specialized metabolites participate in plant ecological interactions by providing protection against biotic and abiotic stresses^1^, ^2^ and promoting environmental adaptation processes.^3^ Bioactive compounds may also have a role in human nutrition, being used as flavor sources, food additives, or medicines, thanks to their health‐promoting properties.^4^

Specialized metabolites are usually classified in groups according to their biosynthetic pathways.^1^ Polyphenols are one of the most abundant groups, highly diversified and subdivided into further subgroups such as phenolic acids, flavonoids, and anthocyanins.^5^ These compounds exhibit antioxidant and anti‐inflammatory properties as well as free radicals scavenging or antimicrobial activities.^6^, ^7^ Carotenoids, another important group of metabolites, not only exhibit important properties for human use and health, but also have photo‐protective roles in plant cells.^8^ Many specialized metabolites such as anthocyanins, betalains, flavonoids, and carotenoids are also responsible for the pigmentation of flowers, fruits, or other plant tissues,^9^ which determine the attraction or repulsion of animals, thus favoring reproductive success and conservation of the species. The biosynthesis and accumulation of specialized metabolites is mainly affected by light‐induced responses.^10^

The ability of plants to respond to light is determined by the presence of multiple photoreceptors, proteins capable of sensing different light intensity, quality, direction, and photoperiod,^11^, ^12^ triggering signals that regulate multiple physiological and metabolic responses.^13^ Five classes of photoreceptors have been identified,^14^ which enable plants to respond to a broad spectrum of light, from ultraviolet B (UV‐B) to far‐red wavelengths.^11^ Phytochromes are the main receptors for red and far‐red light spectral regions (600–750 nm), which are involved in plant growth and development, and also promote seed germination and seedling de‐etiolation.^15^ Conversely, blue wavelengths (400–500 nm) can be absorbed by several photoreceptor classes, namely cryptochromes, phototropins, and zeitlupe.^12^ Cryptochromes, which are also responsible for the absorption of green light,^16^ are involved in plant growth and development and regulate several functions, such as the photoperiodic control of flowering, de‐etiolation, or circadian clock adjustment.^17^ Phototropins, which are also responsive to ultraviolet A (UV‐A) wavelengths (315–400 nm), are primarily involved in plant phototropism, but also mediate fundamental processes such as chloroplast movement and stomatal opening.^18^ Finally, the zeitlupe class is involved in photoperiodic flowering and circadian clock regulation.^12^ Although they are outside the so‐called photosynthetic active radiation (PAR) region (400 to 700 nm), UV‐B wavelengths (280–315 nm) are also absorbed by a specific photoreceptor called UV resistance locus 8 (UVR8),^12^ which is involved in plant survival due to the arrangement of a series of protective gene expression responses.^19^

Besides the morphological and physiological responses described above, photoreceptors play a crucial role in stimulating the biochemical pathways of secondary compounds by modulating the expression of specific genes. The biosynthesis of specialized metabolites is strongly related to the wavelength absorbed by the photoreceptor. For example, blue light photoreceptors have been associated with stimulation of photo‐protective compounds, such as carotenoids and anthocyanins.^20^ It has been hypothesized that this plays a specific role in the feedback regulation of photosynthesis and non‐photochemical quenching (NPQ), an energy‐dissipative response against light stress.^21^, ^22^ On the other hand, phytochrome has been observed to have less impact generally on the production of secondary compounds when subjected to monochromatic red light.^20^ This limit may be associated with the excessive stimulation of the photoreceptor, caused by far‐red unbalancing, with consequences on normal plant development and photosynthesis,^20^, ^23^ probably leading to an uneven distribution of resources among plant metabolic pathways. When plants are exposed to UV‐A radiation, an increase in phenolic compounds, especially flavonoids, as a protective mechanism, may be observed.^24^, ^25^ In the case of UV‐B, the acclimation of plants to stressing conditions may also induce a specific enhancement of some compounds such as tocopherols or glucosinolates.^26^, ^27^ However, specialized metabolite biosynthetic responses to UV exposure can vary widely depending not only on environmental factors (such as temperature) but also on plant genetic features, even within the green and red phenotypes of the same species.^25^, ^28^

The specialized metabolism of plants is mainly triggered by stress conditions. Stress can be related to different factors including temperature, nutrition, or drought.^29^, ^30^, ^31^ Light is also a significant player in the mechanisms of the stress‐induced production of bioactive compounds. Accordingly, plants tend to absorb more energy than necessary for the normal photosynthetic process and carbon fixation. The excess energy must be dissipated efficiently to avoid the production of reactive oxygen species capable of initiating reactions involving radicals that damage proteins, lipids, pigments, or other fundamental bioactive molecules.^21^ However, surplus energy is closely dependent on CO_2_ input, which is, in turn, related to the degree of stomatal opening.^30^, ^31^ The imbalance between the amount of irradiated light and energy consumption due to the limited supply of CO_2_ is the cause of the increased rate of specialized metabolites in a plant. This phenomenon can be observed easily in plants grown in semi‐arid areas, where light, water, and heat stress induce stomatal closure and a consequent higher aromatic value than equivalent plants grown in moderate climates.^30^, ^31^ Plants have developed several methods to dissipate excess energy and produce compounds protecting themselves from photo damage, including the NPQ mentioned above, the re‐oxidation of reduced compounds, and the repair of damaged components.^21^

Nowadays, artificial lighting can enable light features to be controlled, with potential effects on plants' primary and secondary metabolic responses.^23^ Among the diverse artificial lighting systems available on the market, light‐emitting diodes (LEDs) are becoming increasingly popular due to several advantages that make them particularly suitable for horticultural lighting.^32^ These include the possibility to customize the spectral composition according to plant or photoreceptor characteristics, the relatively high efficiency in converting electrical energy to light, long operating lifespan, and possible integration with digital control systems.^32^, ^33^, ^34^ Accordingly, LED lamps are today considered the best option for indoor and vertical farming. These farming systems are characterized by tightly controlled and closed environments that require dynamic adjustment of lighting inputs to achieve the best performance in terms of energy consumption, yield, and product quality.^35^ Concerning qualitative traits of plants, LEDs can affect the biosynthesis of specialized metabolites and antioxidant compounds directly by providing the opportunity to design specific lighting recipes characterized by optimal light spectrum, daily light exposure and intensity.^36^, ^37^ These light features contribute to the development of nutraceuticals and functional foods that promote human health and represent a valuable opportunity for the food industry.^38^

A relevant body of literature has already demonstrated the potential of LEDs to enhance the production of specialized metabolites in various plants categories,^39^, ^40^ although most research traditionally targeted leafy vegetable crops.^41^, ^42^ The present review paper aims to provide a systematic analysis of the most recent research in this field from 2016 to date. It investigates the effect of different indoor LED treatments on the accumulation of specialized metabolites in different species, going beyond vegetable production to focus on valuable species, namely edible flowers, microgreens, medicinal plants, and aromatic herbs.

## MATERIALS AND METHODS

Articles were collected through the Scopus and Web of Science databases, combining specific criteria that include:

1. articles published from 2016 to the present;
2. articles that referred to four crop categories: edible flowers, microgreens, medicinal plants, and aromatic herbs;
3. articles that included LED treatments.

Accordingly, articles that applied other lamp typologies, such as high pressure sodium (HPS) or fluorescent lamps, were excluded from the study. However, if other lamp typologies were used in addition to LED light (e.g. HPS + LED or fluorescent + LED) or only as control treatments, the study was included in the analysis. Moreover, only studies conducted in indoor environments were considered. At the same time, articles that used LEDs as a supplemental lighting sources for natural sunlight were not examined, to focus on indoor and vertical farming possibilities. Furthermore, 14 papers that addressed *in vitro* culture were excluded from the inventory because they presented lab‐scale growing conditions that differed from the horticultural approach commonly applied in indoor farms.

The search was conducted in two phases using specific search strings in order to collect a large number of papers. The first phase consisted of a general search by crop categories (edible flowers, microgreens, medicinal plants, aromatic herbs), using the following search string: ‘crop category name’ *AND* (light emitting diodes *OR* indoor), resulting in, e.g., *microgreens AND (light emitting diodes OR indoor)*. After the preliminary search and the screening of the papers retrieved, the investigation was deepened by selecting the most common species for each crop category and replacing them in the search string, e.g., *Brassica oleracea AND (light emitting diodes OR indoor)*. Once relevant articles were selected, information on lighting treatment characteristics and effects on specialized metabolite content was listed in a table for each crop category (see Table S1 in the supplementary material). Although medicinal and aromatic plants are often considered part of the same crop category, commonly referred to as medicinal and aromatic plants (MAPs),^43^ for the sake of the present study it was preferred to make a clear distinction between the two categories. Medicinal plants were therefore considered to be those plants with a main or unique pharmaceutical and cosmetic function, while aromatic plants are those herbs that are mainly used for flavoring purpose in a general culinary sense, although they have possible medicinal applications. The main information on light treatment included light intensity, light spectrum, and photoperiod. The treatment effect was evaluated for the content of various specialized metabolites, focusing on anthocyanins, carotenoids, phenols, tocopherols, terpenoids, and glycosides. Light treatment features (intensity, spectrum, photoperiod) and phytochemicals (anthocyanins, carotenoids, phenols, tocopherols, terpenoids, glycosides) were evaluated in terms of frequencies to understand the prevalence of cases. With regard to lighting features, given that each study included more than one light treatment with specific light characteristics, each treatment was evaluated as an individual observation. The photoperiod was evaluated grouping the observations into three classes (<12, 12–16, >16 h day^−1^) and the intensity was divided into two classes (<200, ≥200 μmol m^−2^ s^−1^). Spectral frequencies were assessed by counting each time a spectrum occurred individually (red, blue, green, yellow, orange, far‐red, ultraviolet (UV), white) or in combination.

**Figure 1:**
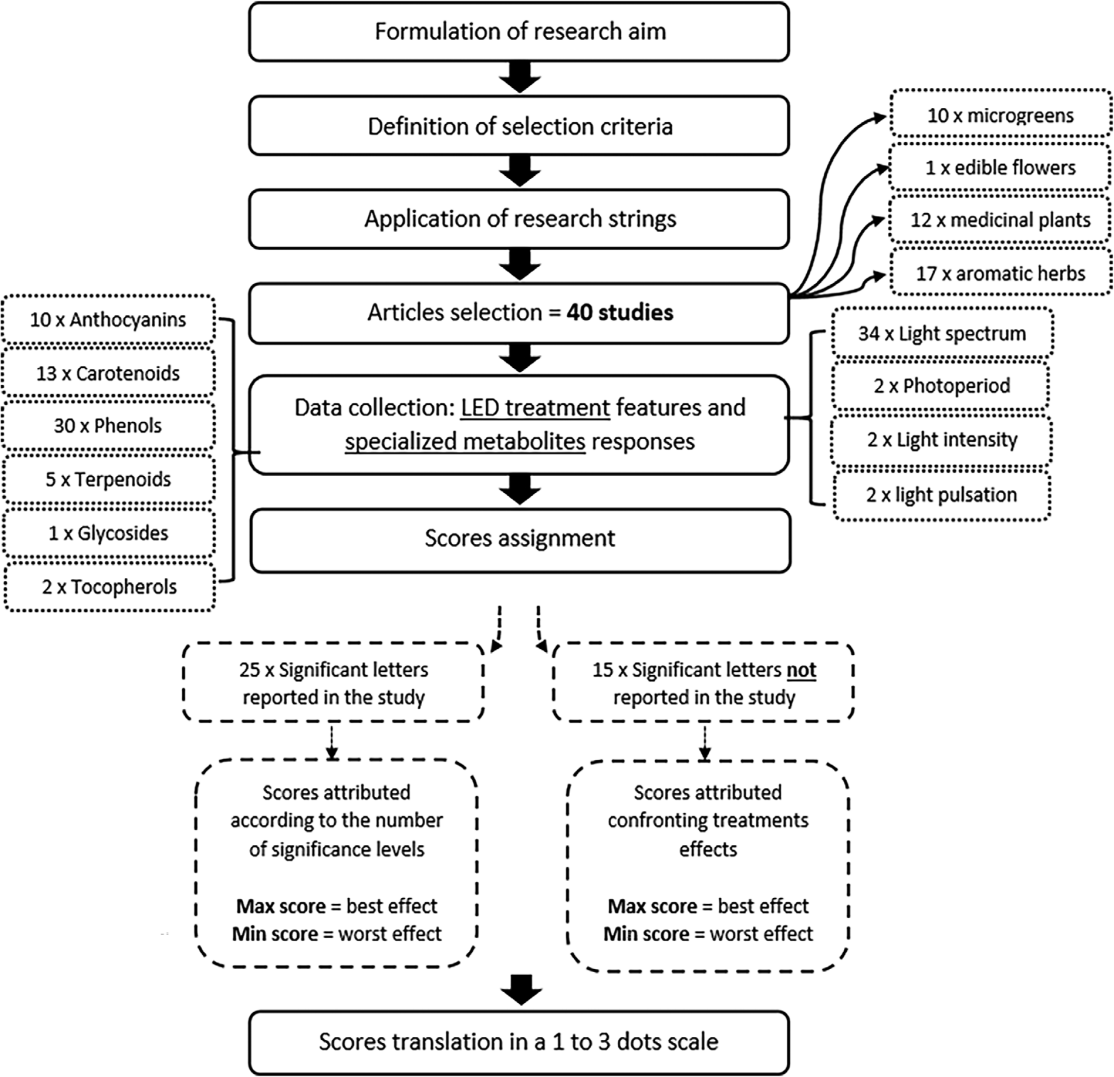
Flow diagram showing the methodological process of review elaboration.

The effects of the different lighting treatments were evaluated within the same study by assigning a score based on the statistical significance of the results. Specifically, the treatment with the highest positive significance received the highest score, and the treatment with the lowest significance received the lowest one. For instance, if a study presented four treatments (T) with three different significance levels (e.g., a, b, c), where T1 = a, T2 = b, and T3 and T4 = c, the best treatment received a score of 3 (T1 = a), the second received 2 (T2 = b), and the third and fourth treatments received 1 (T3 and T4 = c). If the article presented more than three significance levels (e.g., a, b, c, d, e), scores were assigned according to the number of levels (e.g., 1 to 5), with the best result receiving the highest score. In the case of double letters of significance (e.g., ab), the half score was attributed. When all values were assigned, scores were converted to a scale of 1 to 3. Accordingly, for studies with scores higher than 3, the values were normalized, dividing by the highest score assigned and multiplying by 3. For instance, with five significance levels, each value was divided by 1.66 (e.g., divided by 5 and multiplied by 3) to obtain the score 3 for the treatment showing the higher significance. If treatments in a study had no statistically significant differences, all treatments received the maximum score. If an article reported no significance letters but only the average scores of the treatments, the scores were compared with the results of the treatments and classified from best to worst performance. Some studies analyzed multiple compounds for the same specialized metabolite category (e.g., *β*‐carotene, lutein, and neoxanthin for the carotenoids category). In these cases, the final score was assigned by averaging the scores of the compounds analyzed. The intensity of treatment effects on a 1–3 scale is given in the supplementary materials. Figure 1 shows a flow diagram of the methodological process applied to elaborate the review.

## RESULTS AND DISCUSSION

The selection criteria resulted in the selection of 40 papers, ten of which referred to the microgreen crop category, one to edible flowers, 12 to medicinal plants and 17 to the category of aromatic herbs (Fig. 1). Regarding specialized metabolites, phenols were the compounds that were investigated most, being evaluated in 30 articles. Carotenoids and anthocyanins were analyzed in 13 and 10 studies respectively, whereas terpenoids were evaluated in five articles, tocopherols in two articles and glycosides in only one study (Fig. 1). Of the 40 articles, 34 focused on evaluating different spectra, two on photoperiod, two on light intensity, and two on pulsed light (Fig. 1).

**Figure 2:**
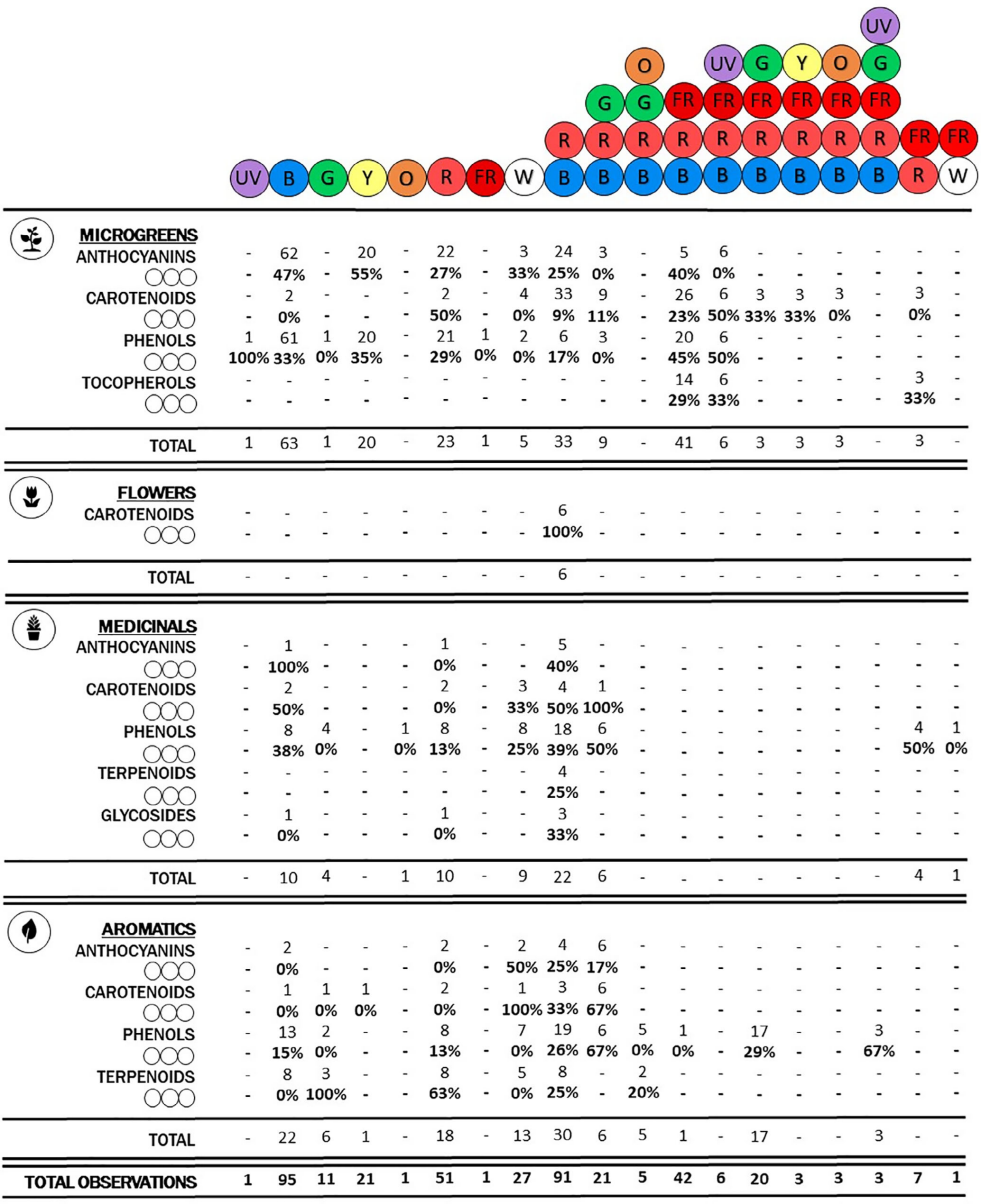
Graphical summary of reviews outcomes showing the absolute frequencies of applied light spectra and percentage of those spectra with a significant positive effect on specialized metabolites (anthocyanins, carotenoids, phenols, tocopherols, glycosides and terpenes) (◯◯◯) in each crop category: microgreens, edible flowers, medicinal plants, and aromatic herbs. (UV = ultraviolet; R = red; B = blue; G = green; Y = yellow; O = orange; FR = far‐red; TOTAL OBSERVATIONS = number of treatments reporting a certain spectrum considering 410 observations).

Among the selected papers, 389 observations or treatments (plus relative controls) reported the photoperiod. Articles that did not report the adopted photoperiod were excluded from the frequency analysis. Most trials applied a photoperiod ranging from 12 to 16 h day^−1^ (n = 369, 95%), with 16 h day^−1^ as the most commonly employed photoperiod (n = 304, 78%). On the other hand, photoperiods lasting more than 16 h day^−1^ or less than 12 h day^−1^, were barely used, occurring 14 (4%) and 6 (1%) times, respectively (Table S1). Light intensity was detailed in 391 observations, excluding control treatments. The majority of observations (n = 301, 77%) employed a light intensity equal to or higher than 200 μmol m^−2^ s^−1^, whereas 90 cases (23%) applied an intensity lower than 200 μmol m^−2^ s^−1^ (Table S1). The intensities ranged from a minimum of 7.3 μmol m^−2^ s^‐1^ ^44^ to a maximum of 330 μmol m^−2^ s^−1^.^45^ Spectral treatments were either monochromatic or occurred in several combinations and ratios. In total, 410 observations were counted for light quality features, both considering monochromatic and combined spectra. Counting each spectrum color separately, red and blue were used most, occurring 253 (34%) and 289 (38%) times, respectively. Other wavelengths were observed to a lesser extent: far‐red n = 85 (11%), green n = 60 (8%), white n = 28 (4%), yellow n = 24 (3%), UV n = 10 (1%), and orange n = 9 (1%) (Table S1 in the supplementary material). Finally, nearly half of 410 observations (47%) applied a combination of red and blue wavelengths (n = 91, 22%) or red and blue combined with other spectra (n = 103, 25%) (Fig. 2). The remaining percentage (53%) mainly applied monochromatic lighting (n = 208, 51%), especially blue, or other polychromatic combinations (n = 8, 2%), not including red and blue (Fig. 2).

In general, red and blue are the most common light spectral regions used in indoor agriculture, often applied in different ratios to achieve higher crop performance.^46^, ^47^ In fact, although a red wavelength is associated with maximum absorption of chlorophyll pigments, blue light induces stomata opening, leading to greater CO_2_ fixation and biomass accumulation,^48^ with optimal consequences on yield. Concerning specialized metabolites, the ability of blue light to induce stomata opening or, conversely, the inability of red wavelengths to induce this effect, may play an interesting role in the modulation of specialized metabolite content. Moreover, the suboptimal amount of CO_2_ due to closed stomata can determine specific stress‐induced metabolic responses in plants, leading to an increase in specialized compounds^30^, ^31^, ^49^ and leading to the possible conclusion that blueless artificial lighting may help to achieve higher accumulations. However, monochromatic red light was observed to have a limited role in specialized metabolites biosynthesis probably due to a phytochrome over‐stimulation linked to lack of far‐red radiation,^20^ highlighting the importance of combining red with other wavelengths, especially blue, to optimize specific metabolisms. Indeed, the phytochrome can also be stimulated by blue light, although with lower efficiency compared to far‐red,^50^ explaining why plants under pure blue light suffer less than those under monochromatic red, which, however, is ineffective in stimulating cryptochrome chain reactions.^51^

When evaluating the relationship between the spectrum and specialized metabolites for each crop category, a large variability among the spectral combinations that optimized nutritional contents was observed (Fig. 2). This consideration can be associated with a species‐specific response across the analyzed crops, and the different reactions of bioactive compounds to light treatment. For instance, anthocyanins may have a higher accumulation under yellow/green/blue light, absorbing these wavelengths to protect chloroplasts,^20^, ^52^ as is clearly visible in Fig. 2 in the microgreens category. In polyphenols such as flavonoids, however, red and blue monochromatic lighting seems to determine an enhancement due to the antioxidant property of these compounds against light‐induced ROS accumulation.^20^, ^53^ Polyphenols, and other bioactive compounds of different crop categories seemed, on the other hand, to be fostered when a combination of red and blue light was applied (Fig. 2). However, generalizations could be equivocal and, therefore, need to be avoided. Accordingly, the following paragraphs report an in‐depth overview of the main results of the different lighting treatments for each crop category, with an effort toward considering each species and each metabolite separately.

### Microgreens

Microgreens are a class of specialty crops characterized by tender and immature greens,^54^, ^55^ which can be obtained from seeds of almost any crop,^56^ including wild species.^57^, ^58^ The edible part of microgreen crops consists of the hypocotyls with cotyledons and first true leaves,^59^ harvested when the cotyledons are fully unfolded and the plant has a minimum height of 5 cm.^60^ The concept of this specialty crop originated in the late 1980s in San Francisco, USA,^54^ and has since been gaining popularity due to its elevated nutritional and sensory properties.^61^ Microgreens are appreciated by consumers for their appearance, taste, and texture,^62^, ^63^, ^64^ and for their high levels of health‐promoting phytochemicals.^62^, ^65^ Microgreens are also classified as ‘functional food’^63^, ^66^ due to their higher content of vitamins, minerals, and bioactive compounds compared with adult plants,^62^, ^63^ and their lower nitrate content.^67^ These characteristics, and the ease with which microgreens can be cultivated, mean that microgreens can be a valuable nutrient‐rich food source even in remote locations with difficult access to fresh vegetables.^68^ Although this crop can be grown in a variety of scenarios, including outdoor, greenhouse, and indoor conditions, cultivation in controlled environments with artificial lighting represents the most common situation.^55^, ^63^ Indoor farming technology is suggested for the production of continuous and uniform high‐quality microgreens using LED light as a specific factor to modify their metabolism and obtain tailored crops rich in bioactive compounds.^48^, ^67^

The wide application and beneficial effects of combined red and blue light seem to be accurate, particularly in the cultivation of microgreens, presenting significant positive responses when applied together and combined with other spectra (Fig. 2). However, the effect of red and blue light on the synthesis of specialized metabolites can vary depending on plant species. Concerning the effects on carotenoids, Lobiuc *et al*.^48^ found that total carotenoid content (TCC) in *Ocimum basilicum* microgreens was not affected by different red and blue ratios compared with white light, as also observed by Meas and colleagues^47^ in *Amaranthus cruentus*. In *B. oleracea* microgreens (Table 1), the carotenoid content increased under a red and blue combination supplemented with far‐red, in contrast with *Brassica rapa*, where carotenoids were increased when a green or yellow irradiation was added to a red, blue, and far‐red spectrum.^56^ However, it should be noted that light quality is not the only parameter that affects TCC. Craver and colleagues^63^ found that TCC in microgreens of *B. rapa* var. Japonica and *Brassica juncea* (Table 1) may also depend on light intensity, showing a lower TCC value when light intensity is increased from 105 to 315 μmol m^−2^ s^−1^. Indeed, it is likely that carotenoid biosynthesis is enhanced at higher light intensities due to their photoprotective functions. However, such effects may be hindered by pigment photodegradation and the dilution effect of increased plant growth,^69^ possibly resulting in a higher concentration of carotenoids in plant tissues exposed to lower light intensities.

**Table 1:** Within‐studies comparison of anthocyanins, carotenoids, phenols and tocopherols content in microgreens of *Brassica* grown under different LED light features (light spectrum, intensity, and photoperiod)

REFERENCES	SPECIES	LIGHTING TREATMENT		SPECIALIZED METABOLITES			
		Standard LED light features	Specific LED light features	Anthocyanins	Carotenoids	Phenols	Tocopherols
Brazaitytė *et al*., 2019^73^	*Brassica juncea* (Red Lion)	10 h day^−1^, 300 μmol m^−2^ s^−1^, R^a^:B^a^:FR^a^	UV^a^‐A (366 nm) = 12.4 μmol m^−2^ s^−1^	◯^b^	◯◯◯^b^	◯◯	◯
			UV (390 nm) = 12.4 μmol m^−2^ s^−1^	◯◯	◯	◯◯	◯◯
			UV (402 nm) = 12.4 μmol m^−2^ s^−1^	◯◯	◯	◯◯◯	◯◯◯
			Standard light (Control)	◯◯◯	◯◯◯	◯	◯◯
	*B. juncea (*Red Lion*)*	16 h day^−1^, 300 μmol m^−2^ s^−1^, R:B:FR	UV‐A (366 nm) = 12.4 μmol m^−2^ s^−1^	◯◯◯	◯	◯◯	◯◯
			UV (390 nm) = 12.4 μmol m^−2^ s^−1^	◯◯◯	◯◯◯	◯◯◯	◯◯
			UV (402 nm) = 12.4 μmol m^−2^ s^−1^	◯◯◯	◯◯◯	◯◯◯	◯◯◯
			Standard light (Control)	◯◯◯	◯	◯◯	◯
Craver *et al*., 2017^63^	*Brassica oleracea* (Gongylodes)	16 h day^−1^, R:B = 87:13	105 μmol m^−2^ s^−1^	◯	◯◯◯	◯◯	
			210 μmol m^−2^ s^−1^	◯◯	◯◯◯	◯◯	
			315 μmol m^−2^ s^−1^	◯◯◯	◯◯◯	◯◯	
	*B. oleracea* (Gongylodes)	16 h day^−1^, R:FR:B = 84:7:9	105 μmol m^−2^ s^−1^	◯	◯◯◯	◯◯◯	
			210 μmol m^−2^ s^−1^	◯◯	◯◯◯	◯◯	
			315 μmol m^−2^ s^−1^	◯◯◯	◯◯◯	◯◯	
	*B. oleracea* (Gongylodes)	16 h day^−1^, R:G^a^:B = 74:18:8	105 μmol m^−2^ s^−1^	◯	◯◯◯	◯	
			210 μmol m^−2^ s^−1^	◯◯	◯◯◯	◯◯	
			315 μmol m^−2^ s^−1^	◯◯	◯◯◯	◯	
	*Brassica rapa* (Japonica)	16 h day^−1^, R:B = 87:13	105 μmol m^−2^ s^−1^		◯◯◯		
			210 μmol m^−2^ s^−1^		◯◯◯		
			315 μmol m^−2^ s^−1^		◯◯◯		
	*B. rapa* (Japonica)	16 h day^−1^, R:FR:B = 84:7:9	105 μmol m^−2^ s^−1^		◯◯◯		
			210 μmol m^−2^ s^−1^		◯		
			315 μmol m^−2^ s^−1^		◯		
	*B. rapa* (Japonica)	16 h day^−1^, R:G:B = 74:18:8	105 μmol m^−2^ s^−1^		◯◯◯		
			210 μmol m^−2^ s^−1^		◯◯◯		
			315 μmol m^−2^ s^−1^		◯◯◯		
	*B. juncea* (Garnet Giant)	16 h day^−1^, R:B = 87:13	105 μmol m^−2^ s^−1^		◯◯◯		
			210 μmol m^−2^ s^−1^		◯		
			315 μmol m^−2^ s^−1^		◯		
	*B. juncea* (Garnet Giant)	16 h day^−1^, R:FR:B = 84:7:9	105 μmol m^−2^ s^−1^		◯◯◯		
			210 μmol m^−2^ s^−1^		◯		
			315 μmol m^−2^ s^−1^		◯		
	*B. juncea* (Garnet Giant)	16 h day^−1^, R:G:B = 74:18:8	105 μmol m^−2^ s^−1^		◯◯◯		
			210 μmol m^−2^ s^−1^		◯		
			315 μmol m^−2^ s^−1^		◯		
Samuolienė *et al*., 2019^56^	*B. rapa* (Japonica)	16 h day^−1^, 300 μmol m^−2^ s^−1^, R:B = 5:1	FR = 4 μmol m^−2^ s^−1^		◯		
			FR = 4 μmol m^−2^ s^−1^, G = 15 μmol m^−2^ s^−1^		◯◯◯		
			FR = 4 μmol m^−2^ s^−1^, Y = 15 μmol m^−2^ s^−1^		◯◯◯		
			FR = 4 μmol m^−2^ s^−1^, O^a^ = 15 μmol m^−2^ s^−1^		◯◯		
	*B. oleracea* (Green)	16 h day^−1^, 300 μmol m^−2^ s^−1^, R:B = 5:1	FR = 4 μmol m‐^2^ s^−1^		◯◯◯		
			FR = 4 μmol m‐^2^ s^−1^, G = 15 μmol m‐^2^ s^−1^		◯◯		
			FR = 4 μmol m‐^2^ s^−1^, Y = 15 μmol m‐^2^ s^−1^		◯		
			FR = 4 μmol m‐^2^ s^−1^, O = 15 μmol m‐^2^ s^−1^		◯◯		
	*B. oleracea* (Delivery purple)	16 h day^−1^, 300 μmol m^−2^ s^−1^, R:B = 5:1	FR = 4 μmol m‐^2^ s^−1^		◯◯◯		
			FR = 4 μmol m‐^2^ s^−1^, G = 15 μmol m‐^2^ s^−1^		◯◯		
			FR = 4 μmol m‐^2^ s^−1^, Y = 15 μmol m‐^2^ s^−1^		◯		
			FR = 4 μmol m‐^2^ s^−1^, O = 15 μmol m‐^2^ s^−1^		◯		
Samuolienė *et al*., 2017^45^	*B. juncea* ^c^ (Red Lion)	16 h day^−1^, R (660 nm) = 170 μmol m^−2^ s^−1^, FR = 2.5 μmol m^−2^ s^−1^	R (638 nm) = 130 μmol m^−2^ s^−1^		◯◯		◯◯◯
			B = 25 μmol m^−2^ s^−1^, R (638 nm) = 105 μmol m^−2^ s^−1^		◯◯		◯◯
			B = 50 μmol m^−2^ s^−1^, R (638 nm) = 80 μmol m^−2^ s^−1^		◯◯		◯◯
			B = 75 μmol m^−2^ s^−1^, R (638 nm) = 55 μmol m^−2^ s^−1^		◯◯◯		◯◯
			B = 100 μmol m^−2^ s^−1^, R (638 nm) = 30 μmol m^−2^ s^−1^		◯◯		◯

^a^ UV = ultraviolet; R = red; B = blue; G = green; Y = yellow; O = orange; FR = far‐red.

^b^ Three dots represent the best performance among all treatments of the same study, one dot represents the worst performance among all treatments of the same study. In case of study treatments with similar effects, three dots were assigned to all treatments.

^c^ Confronted with *Beta vulgaris* and *Petroselinum crispum*.

In contrast with the observations for carotenoids, Lobiuc *et al*.^48^ showed that contents of anthocyanins and phenolic acids in *O. basilicum* microgreens are primarily influenced by the proportion of red and blue. In particular, the content of phenolic acids in a red cultivar (Red Rubin) of *O. basilicum* was higher under a light spectrum consisting of a red:blue ratio (R:B) of 0.5:1. The effectiveness of blue light in increasing phenol content might be determined by a protective mechanism produced by cytochrome P450 against the accumulation of reactive oxygen species (ROS).^70^ On the other hand, anthocyanin content showed the best results under a R:B = 2:1. However, in microgreens of *Brassica oleracea* var. *Gongylodes* (Table 1), the total phenolic content (TPC), seemed to be enhanced when far‐red was added to a red and blue background (R:FR:B = 84:7:9).^63^ Finally, in *A. cruentus* microgreens, red and blue light (R:B = 2.3:1) appeared to increase anthocyanin content compared with monochromatic red or blue light, probably due to the ability of the combined wavelengths to induce the expression of anthocyanin regulatory genes.^48^, ^71^

Monochromatic lighting was, however, shown to increase specialized metabolite content in some microgreen species. Zhang and colleagues^59^ observed that monochromatic blue and UV‐A light could be optimal lighting sources to produce soybean (*Glycine max*) microgreens rich in phenolic compounds. In fact, the absorption of blue and UV‐A light, and the consequent metabolic responses, are regulated by the same receptor, the phototropin,^18^ which has been linked to the production of phenols as photoprotective barriers against excess light exposition and free radicals, mainly when applied monochromatically.^20^, ^72^ Ultraviolet light potentialities were also observed,^73^ with significant increases in carotenoids in *B. juncea* when UV was added to a red, blue and far‐red spectrum (Table 1). Carotenoid biosynthesis seems to be regulated by the physiological response triggered by different blue and UV photoreceptors (e.g., UVR8). In contrast, polyphenol biosynthesis is mainly related to the plant response to UV radiation, acting as antioxidants and UV‐absorbing compounds.^74^ In fact, UV‐A stimulates the expression of UV‐protective genes and the associated accumulation of phenols.^73^ In the work by Brazaitytė and colleagues,^73^ the same trend observed for carotenoids was also observed for tocopherols and phenols, while the anthocyanin content did not show a significant increase after UV addition. Samuolienė *et al*.^45^ suggested a mixed red and far‐red spectrum as the most efficient for enhancing tocopherols in *B. juncea* (Table 1), while in *Beta vulgaris* and *Petroselinum crispum* the highest tocopherol content was associated with a red and far‐red light supplemented with a blue component (resulting in an increase of +16% with R:B = 5:1 and + 33% with R:B = 2:1). Further details of the cases analyzed are reported in Table S1, while Fig. 2 summarizes the main light spectra promoting specialized metabolites in microgreens.

### Edible flowers

In addition to microgreens, edible flowers are another specialty crop that has recently gained interest on the market, as they are appreciated for their appearance and are therefore mainly employed in high‐end cuisine.^75^ In general, the whole flower can be consumed, although only some parts should be used in some species. For instance, in *Tulipa* or *Chrysanthemum*, only the petals are edible, while in *Bellis perennis* only the buds can be consumed.^76^ According to Lu *et al*.,^77^there are 97 families and about 180 species of edible flowers. Beyond being used as ornamental plants, edible flowers also have potential beneficial effects for human health due to their elevated content of specialized metabolites, especially antioxidant compounds.^77^ Several studies investigated various edible flower species as sources of beneficial bioactive compounds from either nutritional or health perspectives, including *Sambucus nigra*, *Cichorium intybus* L., *Hedysarum coronarium*,^78^ *Tropaeolum majus*, *Tagetes erecta*, *Spilanthes oleracea*,^79^ *Antirrhinum majus*, and *Viola wittrockiana*.^80^

Although many studies in recent years have focused on assessing the content of specialized metabolites in edible flowers, only a few studies have investigated the effects of artificial indoor LED lighting on the accumulation of bioactive compounds. In particular, Kopsell *et al*.^81^ aimed to understand the potential enhancement of carotenoids in petal tissue of two different cultivars of *Tagetes tenuifolia* under different light treatments (Table S1). The experimental lighting included a fluorescent treatment and different blue and red LED compositions (R:B in the range 10:90, 20:80, and 40:60).^81^ Results showed that LED lighting treatments positively affected carotenoid content in both cultivars. In particular, carotenoid content in the ‘Lemon Gem’ cultivar had the best performance under R:B = 1:4 (Fig. 2), while lower accumulation was associated with fluorescent lighting.^81^ Moreover, the same positive trend was observed in ‘Tangerine Gem’ cultivar for carotenoid accumulation, although no relevant differences were found specifically for *α*‐carotene, *β*‐carotene and lutein among the different light treatments.^81^

### Medicinal plants

Specialized metabolites are considered to be the key source of pharmaceutical and curative properties of medicinal plants.^3^ The commercial value of these plants is associated with their elevated bioactive compound content,^82^ which determines the effects on human health, including antimalarial, antidiabetic, hepato‐protective, anti‐ulcer, anti‐inflammatory, and antimicrobial properties.^83^ The concentration of specialized metabolites can be improved by growing them in a controlled environment, resulting in lower costs for extraction of the active principles. Furthermore, their facilitated crop management and harvesting suggest a higher profit margin when cultivated in vertical farms.^84^, ^85^ Several studies revealed an association between the accumulation of specialized metabolites and artificial LED treatments in medicinal plants, although they showed different responses depending on species and experimental characteristics (Table S1 and Fig. 2). For instance, *Crepidiastrum denticulatum* is a medicinal plant the extracts of which have anti‐oxidative properties.^86^ According to Park *et al*.,^87^ the *C. denticulatum* phenolic compound content did not show significant differences under monochromatic LED treatments compared with fluorescent light as a control. However, an increased accumulation of phenolic compounds in the shoot was observed under certain spectral compositions (e.g, a R:G:B spectrum with ratios of 8:1:1).^87^ Similarly, Bae *et al*.^86^ analyzed *C. denticulatum* phenolic content in response to different ratios of far‐red combined with red LED light and observed the highest phenolic levels when a red:far‐red (R:FR) ratio between 0.7:1 and 1.2:1 was provided.

*Medicago sativa*, another medicinal plant known for its phyto‐pharmacological potential,^88^ was analyzed by Fiutak *et al*.,^89^ demonstrating a higher accumulation of phenolic compounds and carotenoids under red, green, and blue LED lighting compared with white LED or natural sunlight conditions. Accordingly, these spectral wavelengths play a significant role in plant growth phases, with blue particularly affecting the maturity of chloroplasts and photosynthetic activity.^89^ Similarly, the total amount of phenols and anthocyanins in *Lepidium sativum*, a small medicinal plant belonging to the Cruciferae family, was significantly increased under mixed red and blue LED light compared with the sunlight control.^90^ Thus, it showed an increase in phenol and anthocyanin content by up to +47% and + 33% under R:B = 1.5:1 and R:B = 9:1, respectively. A study on *Digitalis purpurea* also highlighted that a red and blue LED light (R:B = 1:4) increased the carotenoid content, while anthocyanins were instead higher under monochromatic blue and red lights.^91^ The study also investigated glycoside content in *D. purpurea*, where the highest concentrations were observed under R:B = 1:4,^91^ probably due to the role of blue photoreceptors in the biosynthesis and accumulation of these metabolites.^92^ Red and blue light (with R:B of 9:1, 2.3:1, and 0.4:1) also increased the phenol content in *Salvia miltiorrhiza*,^93^ compared with monochromatic white, red or blue LED light. The combination of red and blue light was shown to upregulate the expression of many genes encoding key enzymes in the phenylpropanoid pathway and revealed transcription levels of genes consistent with the accumulation of some phenols compounds (e.g., rosmarinic acid).^93^ Accumulation of carotenoids and phenols were also monitored in *Perovskia* sp.^94^ with an observed increase in carotenoids under a red and blue light (R:B = 2.3:1), whereas phenols were mainly enhanced by monochromatic blue or red lights. It may therefore be advanced that monochromatic light may also become a stress factor for plants affecting the accumulation of metabolites.^95^

Finally, the use of *Astragalus membranaceus* has been investigated in several studies with different LED spectra. Choi *et al*.^44^ cultivated *A. membranaceus* under monochromatic LEDs with different irradiances, and obtained the highest phenol content under the blue treatment at 7.3 μmol m^−2^ s^−1^, although blue light presented the lowest intensity used, as compared with red (28.7 μmol m^−2^ s^−1^), green (24.9 μmol m^−2^ s^−1^), and white (49.8 μmol m^−2^ s^−1^) light. However, while in Choi *et al*.,^44^ white LED proved to be the least efficient in increasing the phenol content of *A. membranaceus*, the same plant showed higher values of phenols under the white treatment than when monochromatic blue and red LEDs were used in Jeong *et al*.,^96^ considering an intensity around 30–40 μmol m^−2^ s^−1^ for all spectra. Similarly, white LED appeared to be most effective in increasing phenol content in *Agastache rugosa* compared to red or blue monochromatic light,^97^ possibly due to the higher penetration ability of the white spectrum within the canopy, leading to increased expression of genes involved in phenylpropanoids biosynthesis.^98^ In Livadariu *et al*.,^99^ different monochromatic LED lights showed similar effects on phenol content in *Cannabis sativa*. This plant was also investigated by Namdar *et al*.,^100^ who applied LED treatment in addition to fluorescent or HPS light throughout the crop cycle (in vegetative or flowering stage). According to these results, R:B = 4:1 treatment during the vegetative stage or for 35 days during flowering mainly increased phenols concentration in inflorescence tissues. Terpenoids in *C. sativa* were also highest when R:B = 4:1 was supplied at the vegetative stage.^100^

### Aromatic herbs

Specialized metabolites play a fundamental role in the aromatic function of several herbs, promoting both the characteristic odors and flavors that are particularly appreciated not only for culinary purposes but also for industrial purposes. The industrial applications of aromatic plants include the production of fragrances and preservatives for the food processing industry, and perfumes and skin products in the cosmetics sector.^101^ Important uses also include pharmaceutical and livestock feeds due to their antioxidant, antimicrobial, and anticoccidial properties favoring both human and animal health.^102^, ^103^

Among the aromatic herbs, sweet basil (*O. basilicum*) is one of the most cultivated, presenting not only a wide application in cooking but also remarkable medicinal use.^104^ Several studies from 2016 to date have examined LED lighting effects on specialized metabolites of different cultivars of basil, and overall confirmed a significant effect of artificial lighting treatments to increase various bioactive compounds (Table 2). According to Lin *et al*.,^105^ cv. ‘Dark opal’ and ‘Caesar’ increased their anthocyanin, phenol, and carotenoid content under an equal combination of red, green, and blue LED light (R:G:B = 1:1:1). Moreover, comparing to ‘Dark opal’, ‘Caesar’ cultivar also demonstrated an enhanced production of phenols and carotenoids by augmenting the red proportion in the LED combination (R:G:B = 4:1:1)^105^ (Table 2). In purple *O. basilicum*, both white LED light and a combination of red and blue (R:B = 2.3:1) significantly boosted the anthocyanin content.^106^ Similarly, R:B = 2.3:1 increased the phenol content in green basil (Table 2). On the other hand, white LED light did not show the same effect for the green cultivar, where a monochromatic blue light was more efficient in fostering phenol content. In fact, no effect of 16 h day^−1^ exposure of blue light for different periods (0 to 48 days) on the phenol content of basil was observed^107^ (Table 2). The authors reported that the selected light intensity for basil in the treatments (300 μmol m^−2^ s^−1^) may also be responsible for the unvaried results and non‐reactive responses, as the light saturation of basil was evaluated considerably higher (e.g., more than 1000 μmol m^−2^ s^−1^).^108^ In Pennisi *et al*.,^46^ the highest phenol concentration in basil in response to red and blue light was associated with R:B = 2:1 or 3:1, supporting the assumption that an increased red component in the mixture may enhance antioxidants production.^109^ In contrast, light intensity (e.g. in the range of 100 to 300 μmol m^−2^ s^−1^) did not increase phenols in basil leaves under red and blue irradiation (R:B = 3:1)^110^ (Table 2). Bantis *et al*.^111^ evaluated the total phenol content under different UV, blue, green, red, and far‐red combinations in two cultivars of *O. basilicum*, ‘Lettuce leaf’ and ‘Red rubin’, and observed the best performances for both cultivars in the case of UV:B:G:R:FR = 1:20:39:35:5. Using a broad spectrum of light with a higher blue percentage could possibly affect phenol production positively, due to stimulation of the PAL enzyme (Phenylalanine Ammonia Lyase), a key enzyme in the phenylpropanoid pathway.^112^ However, the phenol content in the ‘Red Rubin’ cultivar showed relevant enhancements also under increased red and far‐red portions (e.g., UV:B:G:R:FR = 0:14:16:53:17 and 0:12:19:61:8) (Table 2). Finally, Naznin *et al*.^113^ studied the carotenoid accumulation in another basil species, known as Lemon Basil (*Ocimum × africanum*), and observed a considerable increase after application of red and blue light (R:B = 5:1) instead of a monochromatic red light (Table 2).

**Table 2:** Within studies comparison of anthocyanin, carotenoid, phenol, and tocopherol content in *Ocimum basilicum* grown under different LED light features (light spectrum, intensity, and photoperiod)

REFERENCES	SPECIES	LIGHTING TREATMENT		SPECIALIZED METABOLITES			
		Standard LED light features	Specific LED light features	Anthocyanins	Carotenoids	Phenols	Terpenoids
Bantis *et al*., 2016^111^	*O. basilicum* (Lettuce leaf)	14 h day^−1^, 200 μmol m^−2^ s^−1^	UV^a^:B^a^:G^a^:R^a^:FR^a^ = 0:12:19:61:8			◯◯	
			UV:B:G:R:FR = 0:8:2:65:25			◯◯	
			UV:B:G:R:FR = 0:14:16:53:17			◯◯	
			UV:B:G:R:FR = 1:20:39:35:5			◯◯◯^b^	
			Fluorescent (Control)			◯^b^	
	*O. basilicum* (Red rubin)	14 h day^−1^, 200 μmol m^−2^ s^−1^	UV:B:G:R:FR = 0:12:19:61:8			◯◯◯	
			UV:B:G:R:FR = 0:8:2:65:25			◯◯	
			UV:B:G:R:FR = 0:14:16:53:17			◯◯◯	
			UV:B:G:R:FR = 1:20:39:35:5			◯◯◯	
			Fluorescent (Control)			◯◯	
Hosseini *et al*., 2018^106^	*O. basilicum* (Green)	16 h day^−1^, 250 μmol m^−2^ s^−1^	Monochromatic R	◯		◯	
			Monochromatic B	◯		◯◯◯	
			Monochromatic W	◯		◯	
			R:B = 1:1	◯		◯	
			R:B = 2.3:1	◯		◯◯◯	
	*O. basilicum* (Purple)	16 h day^−1^, 250 μmol m^−2^ s^−1^	Monochromatic R	◯◯		◯	
			Monochromatic B	◯◯		◯◯	
			Monochromatic W	◯◯◯		◯◯	
			R:B = 1:1	◯◯		◯◯	
			R:B = 2.3:1	◯◯◯		◯◯	
Lin *et al*., 2021^105^	*O. basilicum* (Dark opal)	12 h day^−1^, 180 μmol m^−2^ s^−1^	R:G:B = 4:1:1	◯	◯	◯	
			R:G:B = 2:1:1	◯◯	◯◯◯	◯◯◯	
			R:G:B = 1:1:1	◯◯◯	◯◯◯	◯◯◯	
	*O. basilicum* (Caesar)	12 h day^−1^, 180 μmol m^−2^ s^−1^	R:G:B = 4:1:1	◯◯◯	◯◯◯	◯◯◯	
			R:G:B = 2:1:1	◯◯◯	◯	◯	
			R:G:B = 1:1:1	◯◯◯	◯◯◯	◯◯◯	
Naznin *et al*., 2019^113^	*Ocimum × africanum* (Lemon Basil)	16 h day^−1^, 200 μmol m^−2^ s^−1^	R:B = 83:17		◯◯◯		
			R:B = 91:9		◯◯		
			R:B = 95:5		◯◯		
			R = 100		◯		
Pennisi *et al*., 2020^110^	*O. basilicum* (Superbo)	16 h day^−1^, R:B = 3:1	100 μmol m^−2^ s^−1^			◯◯◯	
			150 μmol m^−2^ s^−1^			◯◯◯	
			200 μmol m^−2^ s^−1^			◯◯◯	
			250 μmol m^−2^ s^−1^			◯◯◯	
			300 μmol m^−2^ s^−1^			◯◯◯	
Pennisi *et al*., 2019b^46^	*O. basilicum* (Superbo)	16 h day^−1^, 215 μmol m^−2^ s^−1^	R:B = 1:2			◯	
			R:B = 1:1			◯	
			R:B = 2:1			◯◯◯	
			R:B = 3:1			◯◯◯	
			R:B = 4:1			◯	
Rihan *et al*., 2020^121^	*O. basilicum* (Maggie)	16 h day^−1^, 300 μmol m^−2^ s^−1^	R:B = 1:1.5				◯◯
			R:B = 1:1.4				◯◯◯
			R:B = 1:1				◯◯
			Natural light + HPS (Control)				◯
Taulavuori *et al*., 2016^107^	*O. basilicum* (Genovese Gigante)	16 h day^−1^, 300 μmol m^−2^ s^−1^	48 days under enhanced B			◯◯◯	
			36 days under enhanced B			◯◯◯	
			24 days under enhanced B			◯◯◯	
			12 days under enhanced B			◯◯◯	
			0 days under enhanced B			◯◯◯	
Zotov *et al*., 2020^132^	*O. basilicum* (Cinnamom)	14 h day^−1^, 120 μmol m^−2^ s^−1^, 30 days	R:B:G:FR = 33:20:44:3			◯◯	
			R:B:G:FR = 18:57:24:1			◯◯	
			R:B:G:FR = 65:9:23:3			◯◯◯	
			R:B:G:FR = 57:23:18:2			◯◯◯	
	*O. basilicum* (Cinnamom)	14 h day^−1^, 120 μmol m^−2^ s^−1^, 50 days	R:B:G:FR = 33:20:44:3			◯◯	
			R:B:G:FR = 18:57:24:1			◯◯◯	
			R:B:G:FR = 65:9:23:3			◯	
			R:B:G:FR = 57:23:18:2			◯◯	

^a^ UV, ultraviolet; R, red; B, blue; G, green; Y, yellow; O, orange; FR, far‐red.

^b^ Three dots represent the best performance among all treatments of the same study, one dot represents the worst performance among all treatments of the same study. In case of study treatments with similar effects, three dots were assigned to all treatments.

The effect of LED light treatments on specialized metabolite concentration has also been evaluated in other aromatic plants, with consistent beneficial responses (Table S1). For instance, in *Allium fistulosum*, white LED proved to be the most effective treatment for carotenoid accumulation compared with monochromatic light treatments.^114^ In contrast, *Allium sativum* showed a significantly higher accumulation of phenols under monochromatic red light applied with a photoperiod of 16 h day^−1^ compared to monochromatic blue, green and white light.^115^ In the case of *Coriandrum sativum*, monochromatic blue allowed the highest increase in phenol content compared with monochromatic red and green, as well as with red and blue, and red, blue, and far‐red combinations.^116^ Both in *A. sativum* and *C. sativum*, monochromatic green light seems to have a limited effect on plant phenol accumulation. The poor effects of monochromatic green light have already been observed by other authors, although there is insufficient literature investigating this specific wavelength, which prevents general conclusions until there has been further investigation.^20^ In lemon balm (*Melissa officinalis*), phenolic compounds were increased by the simultaneous application of drought stress and a red and blue light (R:B = 2.3:1).^117^ It is possible that the drought stress and the consequent ROS formation^118^ could be balanced by an increase in antioxidant production, particularly stimulated by the R:B treatment.^117^ Alternatively, *Anethum graveolens* presented similar levels of phenol content regardless of the light treatment with different ratios of red, blue, orange, and green light, suggesting that the changes in the red:blue ratios among combinations may have been too narrow to alter phenol accumulation.^119^ However, in the same study, a greater increase in terpenoids occurred when the red component was highest (70% of the spectrum) and the blue component was lowest (10%).^119^ In two *Thymus* species, namely *T. carmanicus* and *T. migricus*, terpenoids were enhanced by monochromatic red light.^120^ Only in *T. migricus*, a combination of red and blue light (R:B = 2.3:1) also increased terpenoid concentration, suggesting that metabolite biosynthesis may be associated with increased stress from red light exposure.^120^ Similarly, a red and blue combination (R:B = 1:1.4, with blue at 435 nm) proved to be efficient in enhancing terpenoid content in basil.^121^ Finally, in *Mentha spicata*, monochromatic red and green were more efficient in increasing terpenoids than monochromatic blue light.^122^ The main light spectra that enhance anthocyanins, carotenoids, phenols, and terpenoids are listed in Fig. 2.

## CONCLUSIONS

This review highlighted the potential of indoor LED treatments to enhance specialized metabolite content in microgreens, edible flowers, medicinal plants, and aromatic herbs. Half of the studies applied a combination of light spectra, which contained red and blue light in different proportions. In some species, lighting combinations, particularly the addition of far‐red or green to the lighting mixture, resulted in an improved synthesis of specialized metabolites. However, in other cases, best performances were also observed under monochromatic red or blue lights. Most trials adopted a photoperiod of between 12 and 16 h day^−1^, often applying 16 h day^−1^ of lighting duration. Among specialized metabolites, more than half of the articles analyzed phenols content, a large number of studies investigated carotenoids and anthocyanins. At the same time, terpenoids, tocopherols and glycosides were evaluated only in few studies. The results of each crop category highlights not only a species‐specific effect of the lighting treatments, but also a different response depending on the analyzed specialized metabolites within the same species. For this reason, giving specific recommendations on the best lighting treatment to be used in each crop category may be equivocal. In general, red and blue light, alone or combined, performed well on different crops and metabolites. However, the application of indoor LED lighting on a commercial scale to enhance product quality should consider the specific responses of different crops and compounds. In this framework, data summarized in Table S1 in the supplementary material may represent a useful tool for producers to identify the best species‐light treatment combination depending on the specialized metabolite to be enhanced.

Beside its commercial usefulness, the present research also aimed to provide suggestions for future research developments. To the best of our knowledge, only one article from 2016 evaluated the phytochemical content in edible flower tissues in response to LED light. Edible flower cultivation using artificial lighting should be further investigated, not only because of the potential nutraceutical improvements that can be achieved with this technology but also because of the high economic value that these products have in their expanding market.^123^ Similarly, although some studies have observed that the application of supplemental UV light enhances bioactive compounds,^59^, ^73^ its application in indoor farming is still under‐researched (mainly due to safety issues and regulations), so further investigation is needed for future sector development. *In vitro* cultivation with LED lighting may also represent an opportunity for the sector, especially for medicinal plants. Moreover, *in vitro* cultivation could also allow for the rapid and large‐scale production of specialized metabolites to be applied in the pharmaceutical and cosmetic industries.^124^, ^125^, ^126^, ^127^, ^128^, ^129^, ^130^, ^131^

## AUTHOR CONTRIBUTIONS

E.A. made contributions to the conception and design, analysis, and interpretation of the data and revised the manuscript critically. I.Z. and L.C. made substantial contributions to the acquisition of data and manuscript drafting. G.P. and F.O. made substantial contributions to the conception and design of the research and critically revised the manuscript. I.P. has critically revised the manuscript. S.Q. and G.P.G. supervised the research and reviewed the manuscript.

## Supporting information


**Appendix S1**. Supplementary Information.
